# The prevalence of five lifestyle risk factors in primary care physicians: A cross-sectional study in Switzerland

**DOI:** 10.1016/j.pmedr.2022.101740

**Published:** 2022-02-19

**Authors:** Liv Mahler, Paul Sebo, Thierry Favrod-Coune, Amir Moussa, Christine Cohidon, Barbara Broers

**Affiliations:** a Institute for Family Medicine and Paediatrics, University of Geneva, Geneva, Switzerland; b Primary Care Division, Geneva University Hospital, Geneva, Switzerland; c Department of Family Medicine, Center for Primary Care and Public Health (Unisanté), University of Lausanne, Lausanne, Switzerland

**Keywords:** Healthy lifestyle, Lifestyle risk factors, Health-related behaviours, Primary care physician, General practitioner, Paediatrician, Gynaecologist, Switzerland

## Abstract

Having a healthy lifestyle is important not only for the health of physicians, but also for the realisation and effectiveness of counselling on patients. Information on lifestyle habits and the presence of health-related behaviours in primary care physicians (PCPs) is lacking.

Using a cross-sectional study design, an anonymous questionnaire was sent to a random sample of 1′000 PCPs practicing in the seven Western cantons of Switzerland. In our sample, we assessed the presence of five lifestyle risk factors, namely current smoking, at risk alcohol consumption, insufficient physical activity, being overweight and insufficient hours of sleep.

510 physicians participated in our study (51% participation rate). Respondents were 51% women, with a majority of general practitioners (67%), followed by paediatricians (19%) and gynaecologists (14%). 57% of PCPs had no or one lifestyle risk factor, 40% had two or three and 3% had four or all five. The average number of lifestyle risk factors was 1.39. Insufficient physical activity was the most prevalent lifestyle risk factor (40%), followed by excess weight and insufficient hours of sleep (32%), at risk drinking (25%) and current smoking (9%). Having ≥2 lifestyle risk factors was associated to being a man, working in a solo practice and for ≥7 half-days per week.

Overall, a majority of Swiss PCPs have no or one lifestyle risk factor, but certain unfavourable health-related behaviours are present, notably insufficient physical activity. Developing strategies and courses to improve physicians’ lifestyles should be proposed early on in the medical curriculum.

## 1 Background

Currently, a “healthy lifestyle” is defined as having sufficient physical activity, a well balanced diet, a Body Mass Index (BMI) within the recommended range, a non or moderate consumption of alcohol, being a non-smoker and obtaining sufficient daily sleep (Znyk et al., 2019, Liu et al., 2016). These six health-related lifestyle factors have often been assessed in the general adult population, and several studies showed that a majority of individuals presented multiple health risk behaviours (Liu et al., 2016, Ford et al., 2012, John et al., 2018 Apr 27, Poortinga, 2007). Although physicians are supposed to recommend healthy behaviours to their patients, they themselves do not always meet these criteria (Wilf Miron et al., 2019, Borgan et al., 2015, Frank et al., 1998, Bakhshi and While, 2013). A recent study carried out in Poland showed that only 11% of general practitioners (GPs) met the criteria for five healthy lifestyle factors assessed (a non or moderate alcohol consumption, a non-smoking status, a BMI within the normal range, a healthy diet, and sufficient physical activity) (Znyk et al., 2019).

Several researchers have noted a higher alcohol consumption among physicians in comparison to the general population (Frank et al., 1998, Romero-Rodríguez et al., 2019, Smith et al., 2017, Sebo et al., 2007, McGrady et al., 2007), with a higher risk of developing dependence to alcohol (Cottler et al., 2013). In a Swiss study carried out in 2002 on 1′784 primary care physicians (PCPs), using the AUDIT-C score the authors found that 30% of responders were at risk drinkers (i.e., approximately twice as much as the general population) (Sebo et al., 2007). However, they also showed that Swiss PCPs were two to three times less likely to smoke (12% active smokers compared to 30% in the general population) (Sebo et al., 2007). Several other studies have reached similar conclusions concerning a lower prevalence of smoking in physicians (Frank et al., 1998, Sebo et al., 2007, McGrady et al., 2007, Juel et al., 1999, Frank, 2000 Oct, Desprès et al., 2010).

With regards to physical activity, most studies showed that physicians were generally active and often more active than the general population for identical age groups (McGrady et al., 2007, Suija et al., 2010, Mandil et al., 2016). Research on the BMI of physicians showed results that varied greatly within countries, ranging from approximately every third physician to more than every second physician being overweight or obese (Znyk et al., 2019, Wilf Miron et al., 2019, Borgan et al., 2015, Desprès et al., 2010, Suija et al., 2010, Ajani, 2004).

According to several recent studies, hours of sleep among physicians averaged between 5.9 h and 6.5 h of daily sleep (Borgan et al., 2015, Most Physicians Sleep Fewer Hours Than Needed For Peak Performance, 2021). Insufficient sleep proved to be associated with decreased performance, increased stress and burnout, and higher risk of obesity, diabetes and hypertension (Smith et al., 2017, Gaba and Howard, 2002, Stewart and Arora, 2019, Reutrakul and Van Cauter, 2018). Evidence showed that the appropriate amount of daily sleep for adults, including physicians, is 7–8 h (Hirshkowitz et al., 2015, Sleep Deprivation and Deficiency, 2021).

Assessing the presence of health-related lifestyle factors among physicians is essential, as healthy behaviours may not only significantly reduce mortality and morbidity (Ford et al., 2012), but also have a notable impact on PCPs’ counselling of their patients (Znyk et al., 2019, Frank, 2000 Oct, Suija et al., 2010, Borgan et al., 2015, Frank et al., 1998, Bakhshi and While, 2013, Kiestra et al., 2020, The Counseling Practices of Internists, 2021, Frank, 2004, Howe et al., 2010, Frank, 2000). For example, PCPs are more likely to address health behaviours and discuss preventive measures if they have integrated them themselves, because they feel qualified and confident in giving these recommendations (Znyk et al., 2019, Borgan et al., 2015).

Due to the lack of recent data on lifestyle risk factors (LRFs) among PCPs, we aimed to assess five key health-related behaviours, i.e. tobacco smoking, alcohol consumption, physical activity, healthy diet and daily sleep among PCPs in Switzerland. In this study, we used the BMI as a surrogate marker for a healthy diet.

## 2 Method

### 2.1 Study design

This cross-sectional study was conducted in November and December 2020 in Western Switzerland. A random, non-stratified sample of 1′000 PCPs (GPs, paediatricians and gynaecologists) working in the seven cantons of Western Switzerland (Geneva, Valais, Vaud, Jura, Neuchâtel, Fribourg and Bern) was asked to complete a questionnaire concerning socio-demographic items and lifestyle risk factors (LRFs) (see below). The PCPs were given the possibility to fill out a paper- or web-based questionnaire, the link to the latter being on the information sheet. Both versions of the questionnaire were identical, and no financial compensation was allocated for participation. One month after having received the invitation, a reminder was sent to all non-responders. This study was approved by the Regional Research Ethics Committee (CCER) (n° 2019-01850) and the questionnaire was pre-tested on a group of five physicians so as to ensure comprehensiveness and acceptability.

## 3 Measures

Five LRFs were assessed in the study: current tobacco smoking, at risk drinking, physical inactivity, being overweight (as a surrogate marker for an unhealthy diet), and insufficient sleep.

Tobacco consumption was assessed through two questions: “In the past 5 years, have you smoked tobacco at least once a day?” (Response options: yes/no) and “On average, how many cigarettes per day do you smoke?” Participants were then classified as non-smokers (answering “no” to the first question and “0” to the second”), current smokers (“yes” to the first question and “≥1” to the second) or former smokers (“yes” to the first question and “0” to the second). Former smokers dating back to more than 5 years ago were classified as non-smokers.

**Fig. 1:**
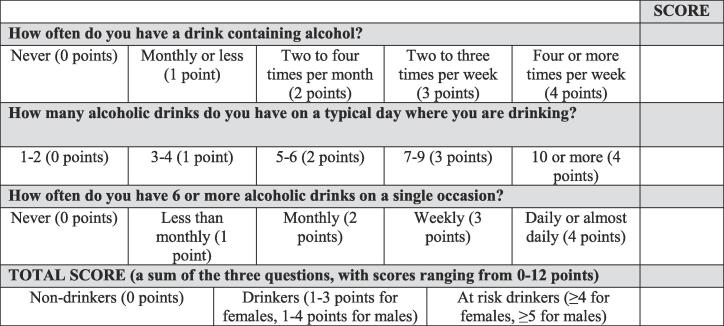
The AUDIT-C score and cut-off scores applied.

Alcohol consumption was evaluated using the 3 AUDIT-C questions: “How often do you have a drink containing alcohol? One alcoholic “drink” corresponding to 10 g of alcohol (1 dl of wine or 2.5 dl of beer)”, “How many alcoholic drinks do you have on a typical day where you are drinking?” and “How often do you have 6 or more alcoholic drinks on a single occasion?” The cut-off scores applied determined three groups: non-drinkers, drinkers, and at risk drinkers (Fig. 1. These cut-offs have shown to be the most effective for identifying individuals in each category (Sebo et al., 2007, Khadjesari et al., 2017, Bush, 1998).

Physical activity was assessed using the following question: “How many times per week do you have a moderate-intensity or vigorous-intensity physical activity (at minimum makes you short of breath or makes you sweat)?” (Global Physical Activity Questionnaire (GPAQ) Analysis Guide [Internet], 2021) Based on this former question and a pre-existing classification from the Swiss Health Survey, a dichotomisation of being sufficiently active (≥2 sessions per week) versus insufficiently active (<2 sessions of exercise per week) was made (3 Déterminants de la santé (Statistiques de la santé), 2019).

BMI was estimated using the height and weight answers provided by our survey, which were then used in the standard calculation of weight in kilograms divided by height in square meters. BMI was then stratified as: underweight (<18.5 kg/m^2^), normal weight (18.5–24.9 kg/m^2^), overweight (25–29.9 kg/m^2^) and obese (≥30 kg/m^2^) (WHO/Europe | Nutrition - Body mass index - BMI [Internet], 2021).

The number of hours of sleep was defined according to the free text question, “On average, how many hours do you sleep per day?” Answers were dichotomised into sufficient (≥7 h) and insufficient hours (<7 h) of daily sleep. This cut-off was defined using the median of our distribution and the recommended hours of daily sleep (Hirshkowitz et al., 2015, Sleep Deprivation and Deficiency, 2021).

### 3.1 Data analysis

The absence or presence of each risk factor was marked as LRF = 0 or LRF = 1, respectively. Presence of LRFs could range from 0 to 5 for each participant. After discussion within the research team we planned to categorize physicians into two groups based on the number of LRFs. Physicians with 0 or 1 LRF were considered to have a healthy lifestyle, whereas those with 2 or more LRFs were considered to have an unhealthy lifestyle.

The prevalence of each LRF was measured using frequency tables. Medians and interquartile ranges (IQR) were used to summarize discrete numerical data as well as continuous numerical data, since the latter did not follow a normal distribution. We tested for trend assuming a linear effect from one category to another using the command ‘contrast’ with the p. operator in STATA. This was also done for variables with more than three categories. Associations between LRFs and socio-demographic characteristics were assessed using chi-squared tests, with a statistical significance set at a value of p ≤ 0.05. All analyses were performed using STATA version 15.1 (College Station, TX, USA).

## 4 Results

Of the 1’000 invitations sent, 510 physicians agreed to participate and filled out our questionnaire (51% participation rate), 73% of participants using the paper-based version. Among responders, 51% were women, and 73% were 45 years of age or older (Table 1). The vast majority of participants were GPs (67%) followed by paediatricians (19%) and gynaecologists (14%). Most respondents were married (70%), worked in duo or group practices (66%), in urban or semi-urban areas (80%) and for a median of 8 half-days per week (IQR 2). Responders were similar to the non-responders regarding gender and working place (55% women, 81% working in urban or semi-urban areas).

**Table 1:** Primary care physicians’ socio-demographic characteristics (N = 510 primary care physicians).

	**N (%)**
**Overall**	510 (100)
**Gender** (N = 510)	
Female	262 (51.4)
Male	248 (48.6)
**Age group** (N = 510)	
<45	140 (27.5)
45–59	229 (44.9)
≥60	141 (27.7)
**Civil status** (N = 508)	
Single	70 (13.8)
Married	358 (70.4)
Divorced/Separated	74 (14.6)
Widowed	6 (1.2)
**Medical speciality** (N = 502)	
General internal medicine	337 (67.1)
Paediatrician	95 (18.9)
Gynaecologist	70 (14.0)
**Type of practice** (N = 502)	
Solo	171 (34.1)
Duo	105 (20.9)
Group	226 (45.0)
**Place of practice** (N = 510)	
Urban	212 (41.6)
Semi-urban	195 (38.2)
Rural	103 (20.2)
**Canton of practice** (N = 510)	
Vaud	170 (33.3)
Geneva	149 (29.2)
Neuchâtel	63 (12.4)
Valais	57 (11.2)
Fribourg	50 (9.8)
Jura	17 (3.3)
Bern	4 (0.8)
**Number of half-days worked per week** (N = 506)	
≤6	122 (24.1)
7–8	189 (37.4)
≥9	195 (38.5)

### 4.1 Smoking status

Of the 510 participants, 90% were classified as non-smokers, 9% as current smokers and 1% as former smokers. Current smoking was more frequent in participants < 45 years old (13%, p-value 0.01) and ≥60 years old (12%, p-value 0.02) compared to those 45–59 years old (5%). Smokers worked least often in duo practices (3%) compared to solo practices (13%, p-value 0.01) and group practices (10%, p-value 0.04). The median number of cigarettes smoked per day was 10 (IQR 13, max 30).

### 4.2 Alcohol use

According to the AUDIT-C cut-off scores, 65% responders were drinkers, 25% at risk drinkers and 10% were non-drinkers. Non-drinking occurred twice as much among women than men (13% and 6% respectively, p-value 0.03).

### 4.3 Level of physical activity

The median number of sessions of physical activity per week was 2 (IQR 2). Overall, 60% respondents reported exercising ≥2 times per week, 28% once a week, and 12% reported not exercising at all. Physicians < 45 years old attained sufficient physical activity less frequently (48%) than those 45–59 (65%, p-value 0.001) and ≥60 years old (63%, p-value 0.01). This followed a statistically significant trend (OR for linear trend 0.73 [95% CI 0.57–0.93], p-value 0.01).

### 4.4 BMI

The median BMI was 23 kg/m^2^ (IQR 4). BMI was in the recommended range for 66% of participants, and 25% and 7% of participants were overweight and obese, respectively. Being overweight or obese was more prevalent among men than women (41% vs. 22%, p < 0.001) and increased with a statistically significant trend across age categories (OR for linear trend 1.86 [95% CI 1.43–2.42], p < 0.001). Physicians working in solo practices had the highest prevalence of excess weight (45%) in comparison to those working in duo practices (24%, p-value 0.001) and group practices (25%, p < 0.001).

### 4.5 Hours of sleep

The median number of hours of sleep was 7 (IQR 1). Recommended sleeping hours (≥7 h) were met by 68% of participants. Paediatricians were more likely to sleep ≥7 h (80%) compared to gynaecologists (59%, p-value 0.003) and GPs (67%, p-value 0.01). Physicians working in solo practices were more likely to sleep < 7 h (40%) compared to physicians in duo practices (20%, p-value 0.001). The prevalence of sleeping < 7 h increased with a statistically significant trend across number of half-days worked per week (OR for linear trend 1.49 [95% CI 1.16–1.91], p-value 0.002).

### 4.6 Combined lifestyle risk factors (LRFs)

Of the respondents, 57% had no or one LRF, 40% had two or three and 3% had four or five (Table 2). The median number of LRFs was 1 (IQR 1). The most prevalent LRF was insufficient physical activity (40%), followed by both insufficient hours of sleep and being overweight (32%), at risk drinking (25%) and current smoking (9%). Having ≥2 LRFs was more prevalent among men than women (49% vs. 37%, p-value 0.01) and among physicians working in solo practices (57%) compared to group practices (38%, p-value 0.001) or duo practices (33%, p < 0.001). Having ≥2 LRFs increased with the number of half-days worked per week (OR for linear trend 1.50 [95% CI 1.18–1.91], p-value 0.001). LRFs were not statistically significant among subgroups of age, civil status, medical speciality or place of practice (Table 3).

**Table 2:** Number of physicians with each of the five lifestyle risk factors (LRFs) investigated in the study.

Lifestyle risk factor (LRF)	N (%)
Insufficient physical activity	205 (40.4)
Insufficient hours of sleep	159 (31.9)
Overweight/obesity	159 (31.5)
At risk drinking	124 (25.4)
Current smoking	47 (9.2)
Number of LRFs^1^	
0	111 (23.2)
1	161 (33.7)
2	133 (27.8)
3	57 (11.9)
4	14 (2.9)
5	2 (0.4)

1 LRFs: current smoking, at risk drinking, overweight/obesity, insufficient physical activity, and insufficient hours of sleep

**Table 3:** Number of physicians with <2 and ≥2 lifestyle risk factors (LRFs) according to socio-demographic characteristics.

	Number of lifestyle risk factors (LRFs) N (%)		p-value^1^
	<2	≥2	
**Overall** (N = 478)	272 (56.9)	206 (43.1)	
**Gender** (N = 478)			**0.01**
Female	154 (62.6)	92 (37.4)	
Male	118 (50.9)	114 (49.1)	
**Age group** (N = 478)			0.29
<45	79 (59.9)	53 (40.2)	
45–59	126 (58.6)	89 (41.4)	
≥60	67 (51.2)	64 (48.9)	
**Civil status** (N = 476)			0.64
Single	39 (59.1)	27 (40.9)	
Married	193 (58.0)	140 (42.0)	
Divorced/Separated	36 (50.0)	36 (50.0)	
Widowed	3 (60.0)	2 (40.0)	
**Medical speciality** (N = 474)			0.26
General internal medicine	182 (56.7)	139 (43.3)	
Paediatrician	55 (63.2)	32 (36.8)	
Gynaecologist	33 (50.0)	33 (50.0)	
**Type of practice** (N = 471)			**<0.001**
Solo	68 (43.3)	89 (56.7)	
Duo	67 (67.0)	33 (33.0)	
Group	133 (62.2)	81 (37.9)	
**Place of practice** (N = 478)			0.79
Urban	112 (56.6)	86 (43.4)	
Semi-urban	99 (55.6)	79 (44.4)	
Rural	61 (59.8)	41 (40.2)	
**Number of half-days worked per week** (N = 477)			**0.003**
≤6	80 (68.4)	37 (31.6)	
7–8	104 (57.5)	77 (42.5)	
≥9	87 (48.6)	92 (51.4)	

1 chi-squared tests.

Current smoking was more often associated with at risk drinking (44%) (p-value 0.003) and physical inactivity (60%) (p-value 0.01) than the absence of this LRF (24% and 38%, respectively). Among at risk drinkers, the proportion of current smokers was higher (16%) than those who were not at risk drinkers (7%) (p-value 0.003). Being overweight was more often associated with inactivity (50%) than being within the normal weight range (36%) (p-value 0.002). Compared to sufficiently active physicians, insufficiently active physicians were more likely to be current smokers (14% vs. 6%, p-value 0.01), have a BMI above the recommended range (39% vs. 26%, p-value 0.002) and sleep < 7 h (39% vs. 27% in sufficiently active participants, p-value 0.01). Finally, participants sleeping insufficient hours were more likely to be insufficiently active (50%) than those sleeping ≥7 h (36%) (p-value 0.01). The strongest association of LRFs was being a current smoker with insufficient physical activity (Table 4).

**Table 4:** Number of physicians with combined lifestyle risk factors (LRFs).

	N (%)	Smoking		p-value^1^	N (%)	Alcohol risk drinking		p-value^1^	N (%)	Overweight or obesity		p-value^1^	N (%)	Inactivity		p-value^1^	N (%)	Sleep insufficiency		p-value^1^
		LRF = 0 N (%)	LRF = 1 N (%)			LRF = 0 N (%)	LRF = 1 N (%)			LRF = 0 N (%)	LRF = 1 N (%)			LRF = 0 N (%)	LRF = 1 N (%)			LRF = 0 N (%)	LRF = 1 N (%)	
**Smoking**				–				**0.003**				0.69				**0.01**				0.51
LRF = 0	463 (90.8)	–	–		443 (90.6)	339 (76.5)	104 (23.5)		458 (90.7)	315 (68.8)	143 (31.2)		461 (90.8)	284 (61.6)	177 (38.4)		451 (90.6)	309 (68.5)	142 (31.5)	
LRF = 1	47 (9.2)	–	–		46 (9.4)	26 (56.5)	20 (43.5)		47 (9.3)	31 (66.0)	16 (34.0)		47 (9.3)	19 (40.4)	28 (59.6)		47 (9.4)	30 (63.8)	17 (36.2)	
**Alcohol risk drinking**				**0.003**				–				0.59				0.32				0.51
LRF = 0	365 (74.6)	339 (92.9)	26 (7.1)		365 (74.6)	–	–		364 (74.6)	246 (67.6)	118 (32.4)		365 (74.6)	214 (58.6)	151 (41.4)		356 (74.3)	246 (69.1)	110 (30.9)	
LRF = 1	124 (25.4)	104 (83.9)	20 (16.1)		124 (25.4)	–	–		124 (25.4)	87 (70.2)	37 (29.8)		124 (25.4)	79 (63.7)	45 (36.3)		123 (25.7)	81 (65.9)	42 (34.2)	
**Overweight or obesity**				0.69				0.59				–				**0.002**				0.13
LRF = 0	346 (68.5)	315 (91.0)	31 (9.0)		333 (68.2)	246 (73.9)	87 (26.1)		346 (68.5)	–	–		346 (68.5)	222 (64.2)	124 (35.8)		340 (68.7)	238 (70.0)	102 (30.0)	
LRF = 1	159 (31.5)	143 (89.9)	16 (10.1)		155 (31.8)	118 (76.1)	37 (23.9)		159 (31.5)	–	–		159 (31.5)	79 (49.7)	80 (50.3)		155 (31.3)	98 (63.2)	57 (36.8)	
**Inactivity**				**0.01**				0.32				**0.002**				–				**0.01**
LRF = 0	303 (59.7)	284 (93.7)	19 (6.3)		293 (59.9)	214 (73.0)	79 (27.0)		301 (59.6)	222 (73.8)	79 (26.3)		303 (59.7)	–	–		296 (59.4)	216 (73.0)	80 (27.0)	
LRF = 1	205 (40.4)	177 (86.3)	28 (13.7)		196 (40.1)	151 (77.0)	45 (23.0)		204 (40.4)	124 (60.8)	80 (39.2)		205 (40.4)	–	–		202 (40.6)	123 (60.9)	79 (39.1)	
**Sleep insufficiency**				0.51				0.51				0.13				**0.01**				–
LRF = 0	339 (68.1)	309 (91.2)	30 (8.9)		327 (68.3)	246 (75.2)	81 (24.8)		336 (67.9)	238 (70.8)	98 (29.2)		339 (68.1)	216 (63.7)	123 (36.3)		339 (68.1)	–	–	
LRF = 1	159 (31.9)	142 (89.3)	17 (10.7)		152 (31.7)	110 (72.4)	42 (27.6)		159 (32.1)	102 (64.2)	57 (35.9)		159 (31.9)	80 (50.3)	79 (49.7)		159 (31.9)	–	–	

## 5 Discussion

### 5.1 Main findings

The results from our survey showed that a majority of Swiss PCPs (57%) live a healthy lifestyle (having no or a single LRF). Having several LRFs was associated with being a man and working in a solo practice, and the number of LRFs increased with the amount of half-days worked per week. Insufficient physical activity was the most prevalent LRF (40%), followed by insufficient hours of sleep (32%), being overweight (32%), at risk alcohol consumption (25%) and being a current smoker (9%).

## 6 Comparison to existing literature

To our knowledge, no other study has evaluated these five LRFs among Swiss PCPs. Our study demonstrated that 9% of physicians were current smokers, showing a 25% relative decrease in the prevalence of smoking among Swiss PCPs since 2002 (12% current smokers) (Sebo et al., 2007). In comparison to the 2017 Swiss Health Survey carried out on the general population, our data suggested that Swiss physicians were three times less likely to smoke than the Swiss general population (9% vs. 27%, respectively) (3 Déterminants de la santé (Statistiques de la santé), 2019). Compared to other studies, the prevalence of current smoking in Swiss physicians is similar to the prevalence reported in Israel (8%) (Wilf Miron et al., 2019), exceeds figures documented in the United States and Northern Ireland (4%) (Frank et al., 1998, McGrady et al., 2007, Frank, 2000 Oct) and is inferior to the prevalence found in Poland (15%) (Znyk et al., 2019) or in France (18% (Desprès et al., 2010) or 32% (Josseran et al., 2005). It is a much lower smoking rate than the one found in a recent meta-analysis, showing 24% smoking among family practitioners (Besson et al., 2021). The results we observed in our study were in concordance with previous research, showing a decrease in the prevalence of smoking tobacco over the years in both physicians and in the general population (Frank et al., 1998, Sebo et al., 2007, McGrady et al., 2007, Juel et al., 1999, Frank, 2000 Oct, Desprès et al., 2010, Besson et al., 2021, Põld and Pärna, 2017, Garfinkel and Stellman, 1986).

We found that 25% of Swiss physicians were at risk drinkers, corresponding to a 5% decrease in absolute terms in comparison to the study carried out in 2002 (30% at risk drinking). Furthermore, we found that 10% of physicians were abstinent, therefore showing that non-drinkers had more than doubled since 2002 (4% abstinence) (Sebo et al., 2007). However, Swiss physicians were less frequently non-drinkers than the general population (18% abstinence) (3 Déterminants de la santé (Statistiques de la santé), 2019). A higher alcohol consumption among physicians compared to the general population has been documented in several studies in various countries (Smith et al., 2017, Sebo et al., 2007, McGrady et al., 2007, Cottler et al., 2013), however the use of heterogeneous screening tests makes comparisons between studies difficult. It has been hypothesized that alcohol is used as a coping mechanism (Smith et al., 2017), which could explain its excessive consumption among physicians.

We found that 60% of Swiss physicians carried out ≥2 sessions of physical activity per week, which is lower than the findings of the Swiss Health Survey for the general population, where 76% of the population achieved ≥2 sessions per week (3 Déterminants de la santé (Statistiques de la santé, 2019). The 40% rate of insufficient exercise in our study was similar to data from Northern Ireland, that showed that 43% of GPs were “physically inactive” (using the International Physical Activity Questionnaire) (McGrady et al., 2007) and superior to a study carried out in Polish GPs that suggested that 32% did not achieve recommended activity levels (150 min moderate-intensity, 75 min vigorous-intensity or 115 min combined-intensity physical activity per week) (Znyk et al., 2019). However, different scoring tools were used. Insufficient exercising could be a result of a lack of time (Smith et al., 2017, Saridi et al., 2019), but must be prioritized, especially given that physical activity is viewed as the most important factor of a healthy lifestyle (Suija et al., 2010).

We also found that 66% of physicians were within the recommended weight range (BMI 18.5–24.9 kg/m^2^), whereas 32% were overweight or obese (BMI ≥25 kg/m^2^). These figures were similar to those of general practitioners in France (Desprès et al., 2010), but more favourable than several studies carried out in other countries (Znyk et al., 2019, Wilf Miron et al., 2019, Borgan et al., 2015, Suija et al., 2010, Ajani, 2004). These results were also better than those described among the Swiss general population (42% overweight/obesity). As shown in the Swiss general population, we found that excess weight was more prevalent among men than women and increased with age (3 Déterminants de la santé (Statistiques de la santé), 2019). Being overweight or obese can be interpreted as a nutrition-related disorder (the consequence of a poor diet) as well as an excessive food intake in comparison to energy expenditure through physical activity. Insufficient exercise among physicians could in part explain the relatively high proportion of overweight persons in our study (Table 4).

We showed that 68% of physicians attained recommended sleeping hours (≥7 h), which is well above a study carried out among physicians in Israel (23%) (Wilf Miron et al., 2019). However, 32% of physicians slept less than the recommended 7 h, with statistically significant less sleep in gynaecologists, in those working in solo practices, and in those working at least 9 half-days per week The average 6.9 h of sleep found were higher than those from existing literature (Performance and Report Says, 2021). Research on the importance of attaining recommended sleeping hours is lacking, even though it has been shown to impact a physician’s practice (Borgan et al., 2015, Stewart and Arora, 2019, Kiestra et al., 2020).

We found that the median number of LRFs was 1 (IQR 1), and that 57% had no or a single LRF, the most prevalent being insufficient physical activity. Having ≥2 LRFs was higher among male doctors working in a solo practice and full-time. There was a cohort effect, where being a male physician working in a solo practice full-time was associated to a higher risk of cumulating LRFs, whereas colleagues who opted for duo or group practices at a part-time base had less LRFs. However, due to the cross-sectional design we cannot conclude on a causal link, but we can suggest that choosing a part-time activity in a duo or group practice may facilitate maintaining a healthier lifestyle. Comparison with other studies assessing lifestyle habits and health-related behaviours of physicians showed that Swiss PCPs have a relatively healthy lifestyle, although direct analogies are difficult to extrapolate due to different lifestyle behaviours being measured and a heterogeneity in assessment tools used (Znyk et al., 2019, Wilf Miron et al., 2019, Borgan et al., 2015, Frank et al., 1998).

Several authors have investigated the negative impact of the medical profession on physicians’ lifestyles, notably the stressful working conditions and the time-consuming nature of the profession, which encroaches on private life (Smith et al., 2017, Lindemann et al., 2019, Nørøxe et al., 2018, Health, 2018, Rosenstein, 2019). In a survey conducted on UK medical graduates, almost half of participants reported that their profession had detrimental effects on their health, with 21% who believed their profession was a cause for their excessive alcohol consumption (used as a coping mechanism) and lack of time, hence leading to poor physical activity and bad eating habits (Smith et al., 2017). Studies have shown that a physician’s own lifestyle is a strong predictor of their counselling (Znyk et al., 2019, Frank, 2000 Oct, Suija et al., 2010, Borgan et al., 2015, Frank et al., 1998, Bakhshi and While, 2013, Kiestra et al., 2020, The Counseling Practices of Internists, 2021, Frank, 2004, Howe et al., 2010, Frank, 2000), and physicians agree that recommending a healthy lifestyle would be more effective if they themselves followed the health recommendations (Znyk et al., 2019). This indicates the importance of informing physicians about the influence their personal lives can have on both their own health and their practice. Physical activity and smoking are the most counselled (Suija et al., 2010, Kiestra et al., 2020), and physicians feel more competent in doing so when they themselves undertake physical activity and are non-smokers. Znyk et al. addressed the importance of being proactive early on in the medical curriculum, with courses on healthy behaviours and their impact on both physicians themselves and their ability to counsel (Znyk et al., 2019).

### 6.1 Strengths and limitations

The strength of this study was the novelty of assessing five LRFs among PCPs. Our study had a number of limitations, the first being the difficulty to generalize results to other countries or even the entirety of Switzerland, given that our sample only concerned physicians practicing in the Western part of Switzerland. Second, no detailed data concerning non-responders was available, therefore a selection bias of participants could not be excluded. Third, data were self-reported, therefore we cannot rule out the presence of a social desirability bias in responses concerning health risk behaviours, such as an excessive alcohol consumption. Furthermore, the questionnaire was based on short validated questions that each had their limitations. Lastly, given the cross-sectional design of the study, the correlations we found do not imply causality.

## 7 Conclusion

Evaluating five lifestyle risk factors among Swiss PCPs showed that a majority presented no or one LRF. Accumulating LRFs was more prevalent among physicians who worked in solo practices and full-time. Tobacco consumption and at risk drinking had both seen a decrease in PCPs over the past two decades, a trend which will hopefully continue in the future. Physical activity was insufficient in PCPs, which could call for specific measures targeting this population. These measures could also potentially impact the prevalence of excess weight. Given that one third of PCPs did not attain recommended sleeping hours, ways how to achieve sufficient sleep should be explored. PCPs can improve the health of the population through their preventive action, counselling and interventions. Regarded as “social models”, their impact can be influenced by their own health-related behaviours. By practicing what they preach, doctors not only improve their own health, but also appear more credible to their patients and more effective in counselling. Implementing interventions and strategies to reinforce physicians’ wellbeing and working conditions as well as providing support tools for improved counselling of patients is primordial, and should be proposed early in the medical curriculum.

## Funding source

This work was supported by the Geneva University Hospitals [grant number DIP MECOM 9531], with no role in the conduct of the research of preparation of the article.

### CRediT authorship contribution statement

**Liv Mahler:** Conceptualization, Methodology, Validation, Formal analysis, Writing – original draft, Writing – review & editing, Funding acquisition, Project administration. **Paul Sebo:** Conceptualization, Methodology, Validation, Formal analysis, Writing – review & editing. **Thierry Favrod-Coune:** Conceptualization, Methodology, Validation, Formal analysis, Writing – review & editing. **Amir Moussa:** Formal analysis, Data curation. **Christine Cohidon:** Conceptualization, Methodology, Validation, Formal analysis, Writing – review & editing. **Barbara Broers:** Conceptualization, Methodology, Validation, Formal analysis, Writing – review & editing.

## Declaration of Competing Interest

The authors declare that they have no known competing financial interests or personal relationships that could have appeared to influence the work reported in this paper.
